# Evaluation of CPK levels during acne treatment with oral isotretinoin^☆^

**DOI:** 10.1016/j.abd.2020.08.020

**Published:** 2021-07-12

**Authors:** Bruna Maggioni Busetti, David Rubem Azulay, Felipe Aguinaga, Edgar Ollague Cordova

**Affiliations:** Instituto de Dermatologia Professor Rubem David Azulay, Santa Casa de Misericórdia do Rio de Janeiro, Rio de Janeiro, RJ, Brazil

Dear Editor,

Isotretinoin is an effective medication in the treatment of acne, which can have adverse musculoskeletal effects. However, factors associated with elevated creatine phosphokinase (CPK) levels are not well established.^1^

A retrospective study was carried out with 63 patients from Instituto de Dermatologia aiming at evaluating the levels of creatine phosphokinase (CPK) in patients using oral isotretinoin (ISO) for the treatment of acne. The parameters assessed in the medical record included the cumulative dose of ISO, weight and body mass index (BMI). The analysis of the correlation between the CPK levels and the variables was performed using Pearson's correlation coefficient.

To assess the correlation between CPK variation and total cumulative dose, the data were plotted on a scatter plot (Fig. 1). Pearson’s coefficient between the two variables was found to be 0.3, which indicates there is a correlation between the cumulative dose of ISO and CPK levels, albeit a weak correlation.

**Figure 1:**
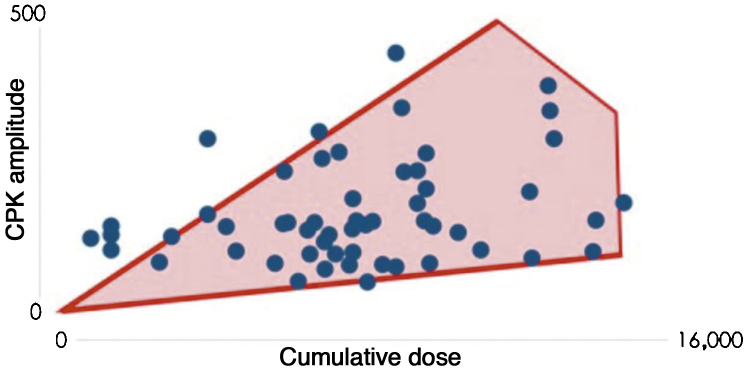
CPK versus total dose of isotretinoin.

Knowing that the cumulative dose varies according to each patient’s weight, a hypothesis was raised that the relationship between the increase in CPK would be associated with the weight and not with the cumulative dose. When correlating the weight with CPK levels (Fig. 2) using Pearson's coefficient, the result is 0.4, showing a moderate correlation in this case, therefore slightly higher than the correlation of the cumulative dose.

**Figure 2:**
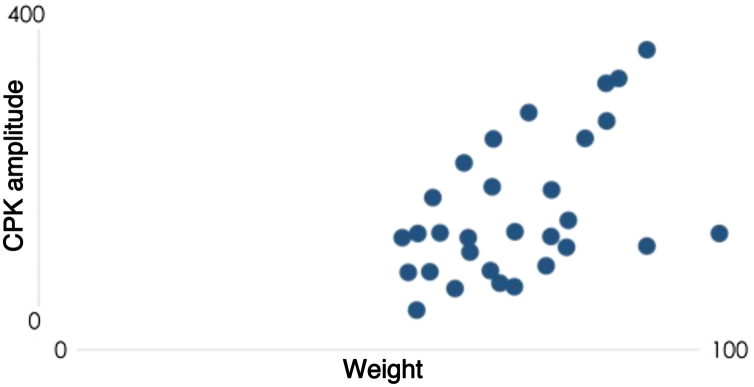
CPK versus weight.

Finally, the correlation between patient BMI and CPK variation was analyzed, since BMI reflects body composition better than weight by itself. Among the studied patients, information was obtained to calculate the BMI in only 30 of them. A moderate positive correlation was also found, with Pearson's coefficient equal to 0.4. When patients were divided into three classes of BMI (underweight, normal weight and overweight) and plotted on the graph shown in Fig. 3, a gradual increase in the maximum CPK value was observed in the group with an increase in the mean BMI.

**Figure 3:**
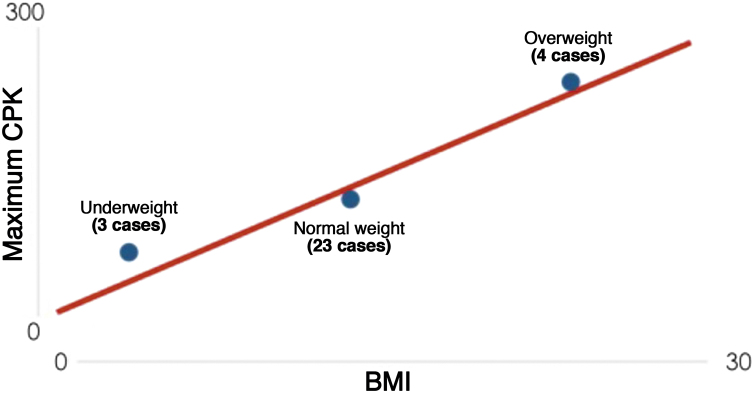
CPK versus mean BMI of the groups.

Recent data show that body composition has been a potent contributor to CPK variability after exercise. Several studies have reported that body fat is a significant factor in muscle damage.^2^ Heled et al. reported that individuals with a higher CPK elevation after exercise have a higher percentage of body fat than those with a lower one.^3^ Paschalis et al. found that overweight women with an average of 31% body fat and 29.5 kg/m^2^ of BMI had a higher CPK elevation after exercise compared to women with normal body weight and BMI.^4^ A study of 119 male high school-age patients divided them into high responders (CPK > 2000 IU/L) and low responders (CPK < 500 IU/L) to CPK increase after exercise, and when the body composition was compared with CPK variation, high responders had a higher percentage of body fat compared to low responders. However, there were no significant differences in muscle mass and BMI between the low and high response groups.^2^ Although these studies were carried out in patients submitted to physical activity, these data reinforce the results of the present study, which suggests that patients with higher weight/BMI are at a higher risk of CPK increase during the use of ISO, unprecedented literature data until now.

Although the relationship between body fat and muscle damage is not fully understood, the possible mechanisms can be inferred from several studies on obesity.^2^ Kriketos et al. reported that individuals with a high percentage of body fat had more type II muscle fibers in biopsied muscles in comparison with non-obese individuals. Type II fibers are known to be more prone to damage than type I fibers. Moreover, obese individuals generally had reduced physical activity. Therefore, obese individuals showed greater muscle damage due to a lesser adaptation.^5^

In conclusion, a correlation was observed between the cumulative dose of ISO and the increase in CPK levels, which are believed to reflect the patients’ weight /BMI, since the latter variables showed a higher level of statistical significance. This finding has already been observed in studies that correlated CPK variations after physical exercise with body composition and BMI. The present study is the first to demonstrate that patients using ISO that have a high BMI may show a higher elevation in CPK levels. Studies with a larger number of patients are required to confirm this association.

## Financial support

None declared.

## Authors’ contribution

Bruna Maggioni Busetti: Statistical analysis; design and planning of the study; drafting and editing of the manuscript; collection, analysis and interpretation of data.

David Rubem Azulay: Effective participation in research orientation; critical review of the manuscript.

Felipe Aguinaga: Approval of the final version of the manuscript; design and planning of the study; drafting and editing of the manuscript; collection, analysis and interpretation of data; effective participation in research orientation.

Edgar Ollague Cordova: Drafting and editing of the manuscript; collection, analysis and interpretation of data.

## Conflicts of interest

None declared.
